# Comparison of dual-energy computer tomography and dynamic contrast-enhanced MRI for evaluating lung perfusion defects in chronic thromboembolic pulmonary hypertension

**DOI:** 10.1371/journal.pone.0251740

**Published:** 2021-06-17

**Authors:** Tawfik Moher Alsady, Till F. Kaireit, Lea Behrendt, Hinrich B. Winther, Karen M. Olsson, Frank Wacker, Marius M. Hoeper, Serghei Cebotari, Jens Vogel-Claussen

**Affiliations:** 1 Institute of Diagnostic and Interventional Radiology, Hannover Medical School, Hannover, Lower Saxony, Germany; 2 Biomedical Research in End-Stage and Obstructive Lung Disease (BREATH), German Center for Lung Research, Hannover, Lower Saxony, Germany; 3 Department of Respiratory Medicine, Hannover Medical School, Hannover, Lower Saxony, Germany; 4 Department of Cardiothoracic, Transplantation and Vascular Surgery (HTTG), Hannover Medical School, Hannover, Lower Saxony, Germany; Medical University of Vienna, AUSTRIA

## Abstract

**Objectives:**

To evaluate the agreement in detecting pulmonary perfusion defects in patients with chronic thromboembolic pulmonary hypertension using dual-energy CT and dynamic contrast-enhanced MRI. Second, to compare both imaging modalities in monitoring lung perfusion changes in these patients after undergoing pulmonary endarterectomy.

**Methods:**

20 patients were examined with CT and MRI before and/or after pulmonary endarterectomy. Estimated perfusion defect percentage from both modalities was compared in a lobe-based analysis. Spatial agreement of perfusion defect maps was also assessed.

**Results:**

A significant correlation between CT and MRI based perfusion defect percentage was calculated in all lung lobes (r > 0.78; p < 0.001). In addition, a good spatial agreement between perfusion defect maps was found (mean spatial overlap for the whole lung was 68.2%; SD = 6.9). Both CT and MRI detected improvements in pulmonary perfusion after pulmonary endarterectomy: 8% and 7% decrease in whole lung perfusion defect percentage (p = 0.007 and 0.004), respectively. In a lobe-wise analysis, improvements were statistically significant only in lower lobes using both modalities (reduction in defect percentage ranged from 16–29%; p < 0.02).

**Conclusions:**

Dual-energy CT is an alternative to MRI in monitoring chronic thromboembolic pulmonary hypertension. Both imaging modalities provided comparable estimations of perfusion defects and could detect similar improvement in lung perfusion after pulmonary endarterectomy.

## Introduction

Chronic thromboembolic pulmonary hypertension (CTEPH) is a subgroup of pulmonary hypertension characterized by obliteration or occlusion of pulmonary arteries due to post-embolic fibrotic material [1]. It subsequently leads to right heart failure if not adequately treated [2]. CTEPH is underdiagnosed [3] due to lack of specific symptoms or misclassification as an acute pulmonary embolism [4]. Pulmonary endarterectomy (PEA) is an effective and in about 70% potentially curative therapy with low perioperative mortality [5, 6]. PEA is currently considered the first-choice therapy in operable patients [7, 8].

Besides lung scintigraphy, dynamic contrast-enhanced MRI (DCE-MRI) is an established radiation-free utility for assessing regional lung perfusion for the purposes of screening and above all monitoring CTEPH patients [9, 10]. Previous studies on CTEPH patients who underwent PEA or BPA could show a good correlation between pulmonary perfusion parameters from DCE-MRI and improvement in clinical and cardiac function [11, 12]. Dual-energy computed tomography (DECT) has also been used for the quantification of pulmonary blood volume (PBV) in patients after pulmonary embolism and in diagnosed CTEPH [13–15]. A previous study [16] utilized DECT in assessing therapy effects after BPA in CTEPH patients. The authors found significant correlations between calculated PBV changes and improvements in 6-min walking distance, mean pulmonary artery pressure (mPAP), cardiac index and pulmonary vascular resistance. Both DECT and DCE-MRI correlate well with perfusion scintigraphy in patients with suspected CTEPH as the current clinical standard [15, 17, 18]. However, how DECT and DCE-MRI correlate with each other in CTEPH patients undergoing PEA is currently unknown. Although CT has the disadvantage of radiation exposure, it offers a higher spatial resolution than MRI, is more practical than MRI (especially in postoperative settings due to shorter examination duration) and it could be used in patients with contraindications to MRI. MRI is however advantageous in the assessment of pulmonary perfusion dynamics and right heart function.

The aim of this work is to compare DECT and DCE-MRI in CTEPH patients, and to compare both imaging modalities in monitoring perfusion changes in the lung parenchyma after PEA.

## Materials and methods

### Patient population

The local ethics committee at Hannover Medical School has approved the study. All patients with CTEPH who were referred to the clinic of cardiothoracic and vascular surgery for PEA were invited to participate in this study. Exclusion criteria were contraindications to CT contrast media (among others, known allergy, renal failure and hyperthyroidism) or MRI (e.g., incompatible metal implants). Between April 2018 and August 2019 twenty-one patients were consecutively included (56.1 ∓ 13.5 years old, 11 males, 10 females). One patient was retrospectively excluded due to incomplete, non-usable imaging. Written informed consent was obtained from all participants.

DECT and DCE-MRI scans were acquired pre- and post-op. The interval between pre-op imaging and PEA was 4.1 ± 5.4 days for CT and 3.7 ± 4.8 days for MRI. The interval between PEA and post-op imaging was 9.6 ± 3.4 days for CT and 8.9 ± 2.8 days for MRI. The interval between CT and MRI scans was pre-op 0–1 days (mean 0 ± 0.5) and post-op 0–2 days (mean 0.3 ± 0.7). 7 patients completed all four scan sessions. Completed pre- and post-op scan pairs per modality were 12 CT and 11 MRI pairs. Overall, 19 patients had both CT and MRI scans for at least one of the scan phases (pre- or post-op).

### DECT protocol and CT-PBV maps reconstruction

The acquisitions were performed on a dual-source CT-scanner (SOMATOM Force, Siemens Healthcare). A test bolus slice was scanned with intravenous injection of 10 ml contrast medium (400 mg iodine/ml; Imeron 400 MCT, Bracco Imaging) followed by 30 ml of normal saline at 4 ml/s. Time to peak (TTP) opacification in the left ventricle was identified from the test bolus time-density curve and set as a fixed delay for the following main acquisition. This timepoint was chosen according to the work from Masy et al. [15] so that lung parenchyma is expected to have an optimal contrast enhancement. The DECT scan was obtained using 150Sn/90 kVp and 0.6 mm collimation with injection of 50 ml contrast medium followed by 50 ml of normal saline flush at 4 ml/s. The tube current was modulated as a function of angle and position along the Z-axis (CARE Dose 4D, Siemens Healthcare). Scan direction was caudocranial with coverage from diaphragm to lung apex in held gentle inspiration (to avoid Valsalva maneuver). Reconstructions used a 1 mm slice thickness with a QR40 convolution kernel.

Lung PBV quantification (derived iodine maps) was performed using syngo.CT DE Lung Analysis (syngo Multimodality Workplace, Siemens Healthcare). In order to minimize noise artefacts, a 3D Gaussian-smoothing filter (standard deviation of 2) was applied to CT-PBV maps using MATLAB (R2018b, MathWorks).

### DCE-MRI protocol for the quantification of pulmonary blood flow (MRI-PBF) and volume (MRI-PBV)

The acquisitions were performed on 1.5T scanners (MAGNETOM Aera and Avanto, Siemens Healthcare). For each patient, the post-op scan was scheduled to take place on the same scanner as pre-op. First, anatomical images of the lung were acquired, including a coronal T2-weighted sequence (half fourier single-shot turbo spin-echo; HASTE) and a transversal steady-state coherent sequence (true fast imaging with steady state precession; TrueFISP). In addition, cardiac imaging with a four-chamber view cine and a short axis cine from the valvular region to the apex was performed using a retrospective gated TrueFISP sequence. Lastly, a 3D time-resolved angiography with stochastic trajectories (TWIST) sequence was acquired in a single breath-hold at end inspiration with the following parameters: FoV 276 x 340 mm^2^–421 x 500 mm^2^, matrix size 192 x 113–256 x 146, slice thickness 3 mm—6 mm, TE 0.7 ms—0.89 ms, TR 2.13 ms—2.74 ms, flip angle 10° - 30°, bandwidth 570 Hz/px—744 Hz/px, temporal resolution 0.8 s—1.1 s. A bolus of 0.04 mmol/kg bodyweight of gadoteric acid (0.08 ml/kg Dotarem, Guerbet) was injected at a rate of 4 ml/s followed by 30 ml of normal saline flush.

PBF maps (pulmonary perfusion in blood flow ml per minute and 100 ml) were calculated according to a deconvolution algorithm [19] with discretization using the Volterra formula, which provides accurate results for a small amount of dispersion and delay of the estimated arterial input function. For that, a self-developed MATLAB script was used. In addition, PBV was calculated according to the central volume theorem as the integral of the residual function.

To assess right cardiac function, cardiac cine images were analyzed by a trained radiology resident (3 years of cardiopulmonary imaging experience, T.M.A.) using CVI^42^ (Circle Cardiovascular Imaging).

### Lung segmentation and co-registration

The segmentation of lung lobes was automatically generated for CT data sets using a further development of a previously trained neural network [20] that was derived from the 3D U‐Net architecture [21] and implemented in TensorFlow [22]. Resulting segmentations were controlled and if necessary, manually corrected by a senior radiology resident (T.M.A.) using ITK‐Snap [23]. Segmentations of MRI TWIST data sets were generated by a nonlinear-registration of the corresponding CT segmentation masks (same patient and same imaging period). In few cases where the patient did not get a corresponding CT scan in the same imaging period (pre- or post-op), the patient’s CT segmentation from the other imaging period was used for the registration. The first 3D volume of the TWIST data set was set as the reference image for the registration. The registrations were performed using the Advanced Normalization Tools (ANTs) software [24]. Resulting MRI segmentations were also visually controlled by a senior radiology resident (T.M.A.).

### Perfusion defect maps and percentage (QDM and QDP, respectively)

A threshold was applied to preprocessed CT-PBV, MRI-PBV and MRI-PBF maps in order to generate QDM and calculate a QDP for each lung lobe and for the whole lung. An independent threshold for each patient, imaging modality, scan session, and perfusion parameter was defined as follows: 75th percentile of lung PBV or PBF x 0.7. In lobe-based analysis, the reference for calculating QDP was the lobe volume as measured from the segmentation mask. An additional calculation of lobar QDP in reference to whole lung volume was done to estimate the contribution of each lobe to the whole lung QDP. For clarity, QDP / QDM calculated from MRI-PBV and from MRI-PBF will be abbreviated in following text as MRI_(PBV)_-QDP / MRI_(PBV)_-QDM and MRI_(PBF)_-QDP / MRI_(PBF)_-QDM, respectively. QDP / QDM calculated from CT-PBV will be mentioned as CT- QDP / CT-QDM.

### Statistical analysis

Lobe-based correlation analyses (Pearson product-moment correlation) between CT-QDP and MRI_(PBF)_-QDP as well as between CT-QDP and MRI_(PBV)_-QDP were done on 19 data sets with completed CT and MRI scan pairs. For the 7 patients who completed all scan sessions, only one random session was included to avoid bias (5 pre- and 2 post-op sessions were included). In order to assess the spatial agreement of the detected perfusion defects, a spatial overlap metric and Dice coefficients for defect and healthy labels were calculated on a voxel level between CT-QDM and MRI_(PBF)_-QDM as well as between CT-QDM and MRI_(PBV)_-QDM. For that, a co-registration between CT and MRI images was achieved by applying the transformation fields from the previously described registration of the segmentation masks. The results of the registration were controlled by a senior radiology resident (T.M.A.). The overlap metric was calculated as follows: overlap = sum of matching defect and healthy voxels / number of all lung (or lobe) voxels.

The second part of the analysis was assessing changes in pulmonary perfusion defects after PEA. Lobe-based paired t-tests of pre- and post-op CT-QDP, MRI_(PBF)_-QDP and MRI_(PBV)_-QDP were performed for patients with completed CT scans (pre- and post-op, n = 12) and for patients with both MRI scans (n = 11).

For all mentioned analysis steps, a significance level of 0.05 was used. The analyses were performed using MATLAB (R2018b, MathWorks).

## Results

### Correlation between DECT and MRI estimations of lung perfusion defects

**Fig 1:**
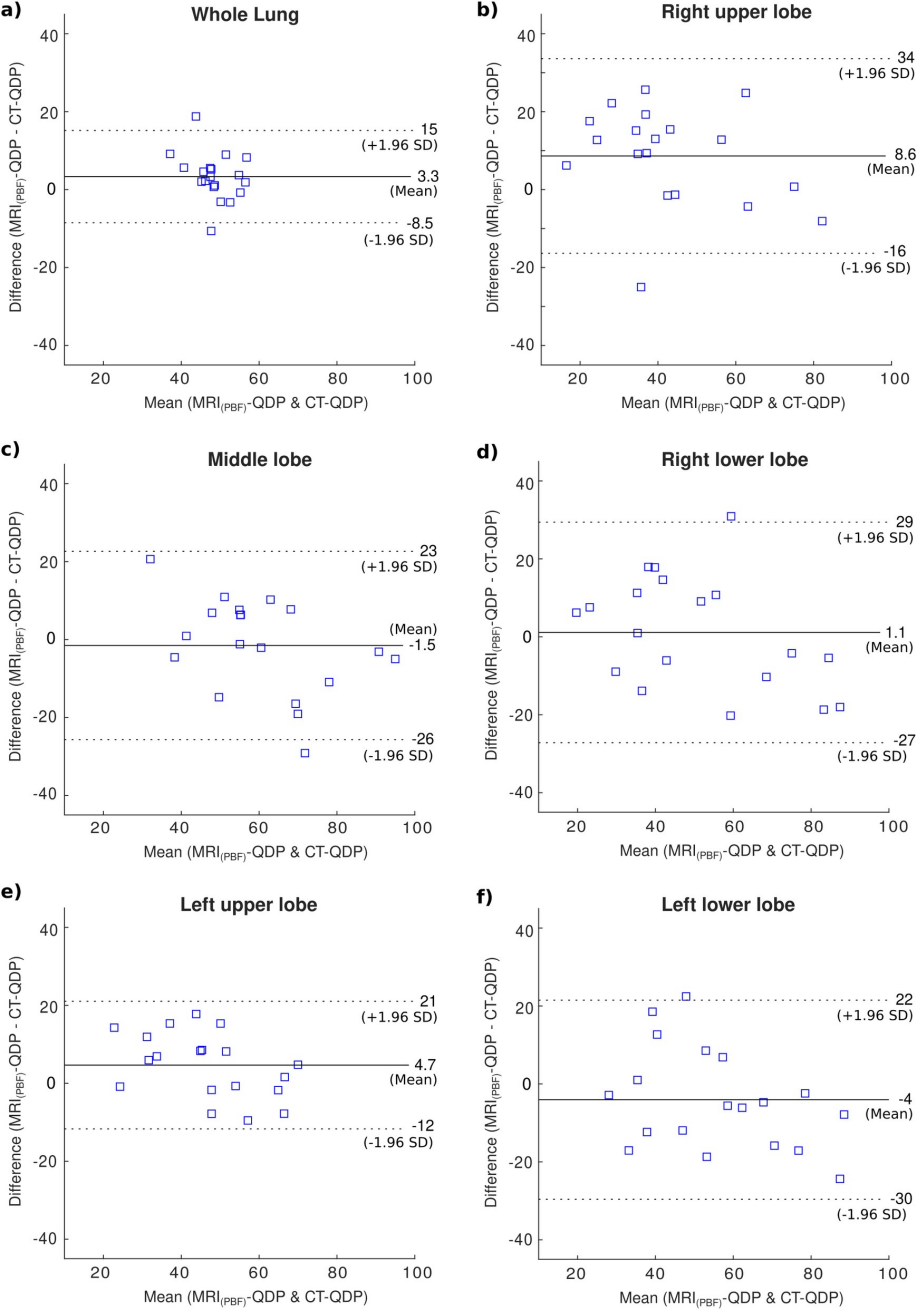
Bland-Altman plots of CT-QDP and MRI_(PBF)_-QDP in 19 patients. (a) QDP of the whole lung. (b-f) Lobe based calculations.

**Fig 2:**
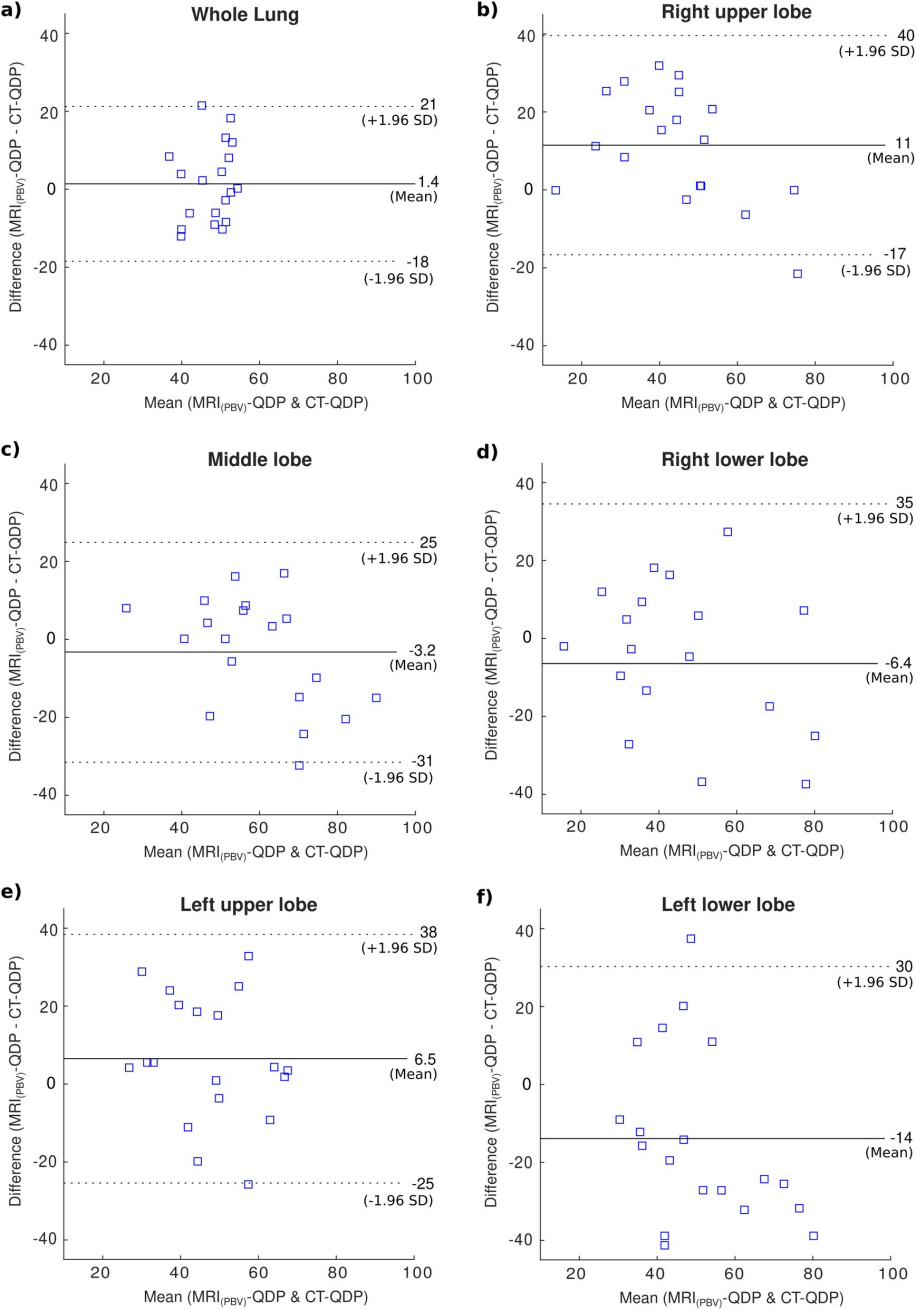
Bland-Altman plots of CT-QDP and MRI_(PBV)_-QDP in 19 patients. (a) QDP of the whole lung. (b-f) Lobe based calculations.

In all lung lobes, similar and significantly correlated QDP values were estimated when using CT-PBV and MRI-PBF (r > 0.78; p < 0.001; Table 1 and Fig 1), and in case of CT-PBV with MRI-PBV significantly correlated for only right lung lobes (right lung lobes r > 0.56; p < 0.012, other lobes r < 0.28; p > 0.23; Table 2 and Fig 2). Mean CT-QDP, MRI_(PBF)_-QDP and MRI_(PBV)_-QDP for the whole lung were 47% (SD 7%), 50% (SD 5%) and 48% (SD 8%), respectively. Mean overlap of whole lung CT-QDM and MRI_(PBF)_-QDM was 68% (SD 7%) while mean overlap between CT-QDM and MRI_(PBV)_-QDM was 62% (SD 8%).

Correlations between absolute CT-PBV and MRI-PBF values did not reach statistical significance in any lung lobe (r < 0.44; p > 0.06, see S1 Table). The correlations between CT-PBV and MRI-PBV values were also not statistically significant in any lung lobe (r < 0.41; p > 0.08).

**Table 1:** Correlation between CT-QDP and MRI_(PBF)_-QDP as well as spatial overlap metrics between CT-QDM and MRI_(PBF)_-QDM. n = 19 patients.

ROI	CT-QDP (%)	MRI_(PBF)_-QDP (%)	Pearson correlation coefficient		QDM overlap (%)	QDM Dice coefficient (Defect)	QDM Dice coefficient (Healthy)
			r	p-value			
**Whole lung**	47 (7)	50 (5)	0.51	**0.026**	68 (7)	0.67 (0.09)	0.69 (0.07)
**Right upper lobe**	39 (20)	47 (17)	0.78	**<0.001**	69 (7)	0.59 (0.14)	0.68 (0.17)
**Right middle lobe**	61 (20)	60 (15)	0.79	**<0.001**	67 (10)	0.69 (0.15)	0.52 (0.18)
**Right lower lobe**	50 (25)	51 (19)	0.82	**<0.001**	69 (9)	0.64 (0.15)	0.60 (0.23)
**Left upper lobe**	45 (17)	49 (13)	0.87	**<0.001**	67 (7)	0.61 (0.15)	0.66 (0.11)
**Left lower lobe**	58 (22)	54 (17)	0.80	**<0.001**	68 (10)	0.68 (0.16)	0.56 (0.24)

Listed values are means with corresponding standard deviations in brackets. Bold p-values denote statistical significance (α = 0.05). CT-QDP and MRI_(PBF)_-QDP: perfusion defect percentage estimated from dual-energy CT (iodine PBV) and DCE-MRI (using the PBF parameter), respectively. QDM: perfusion defect map.

**Table 2:** Correlation between CT-QDP and MRI_(PBV)_-QDP as well as spatial overlap metrics between CT-QDM and MRI_(PBV)_-QDM. n = 19 patients.

ROI	CT-QDP) %(	MRI_(PBV)_-QDP) %(	Pearson correlation coefficient		QDM overlap (%)	QDM Dice coefficient (Defect)	QDM Dice coefficient (Healthy)
			r	p-value			
**Whole lung**	47 (7)	48 (8)	0.08	0.744	62 (8)	0.60 (0.09)	0.64 (0.08)
**Right upper lobe**	39 (20)	50 (14)	0.71	**0.001**	63 (10)	0.54 (0.17)	0.63 (0.16)
**Right middle lobe**	61 (20)	58 (13)	0.70	**0.001**	64 (9)	0.67 (0.15)	0.49 (0.18)
**Right lower lobe**	50 (25)	44 (18)	0.56	**0.012**	64 (9)	0.57 (0.16)	0.59 (0.23)
**Left upper lobe**	45 (17)	51 (14)	0.44	0.062	61 (8)	0.56 (0.14)	0.60 (0.14)
**Left lower lobe**	58 (22)	44 (15)	0.28	0.238	60 (9)	0.58 (0.14)	0.54 (0.23)

Listed values are means with corresponding standard deviations in brackets. Bold p-values denote statistical significance (α = 0.05). CT-QDP and MRI_(PBV)_-QDP: perfusion defect percentage estimated from dual-energy CT (iodine PBV) and DCE-MRI (using the PBV parameter), respectively. QDM: perfusion defect map.

### Cardiovascular and pulmonary perfusion after PEA

All patients had a significant decrease in mPAP measured post-op (pre-op mPAP 42.6 ± 8.4 mmHg, mean decrease 18.8 ± 9.4 mmHg, paired t-test p-value < 0.0001). A significant increase in RVEF was also measured from MRI cine sequences in all 12 patients who completed both MRI acquisitions (RVEF pre-op 38.5 ± 10.9%, RVEF increase 7.1 ± 7.9%, paired t-test p-value < 0.0001).

**Fig 3:**
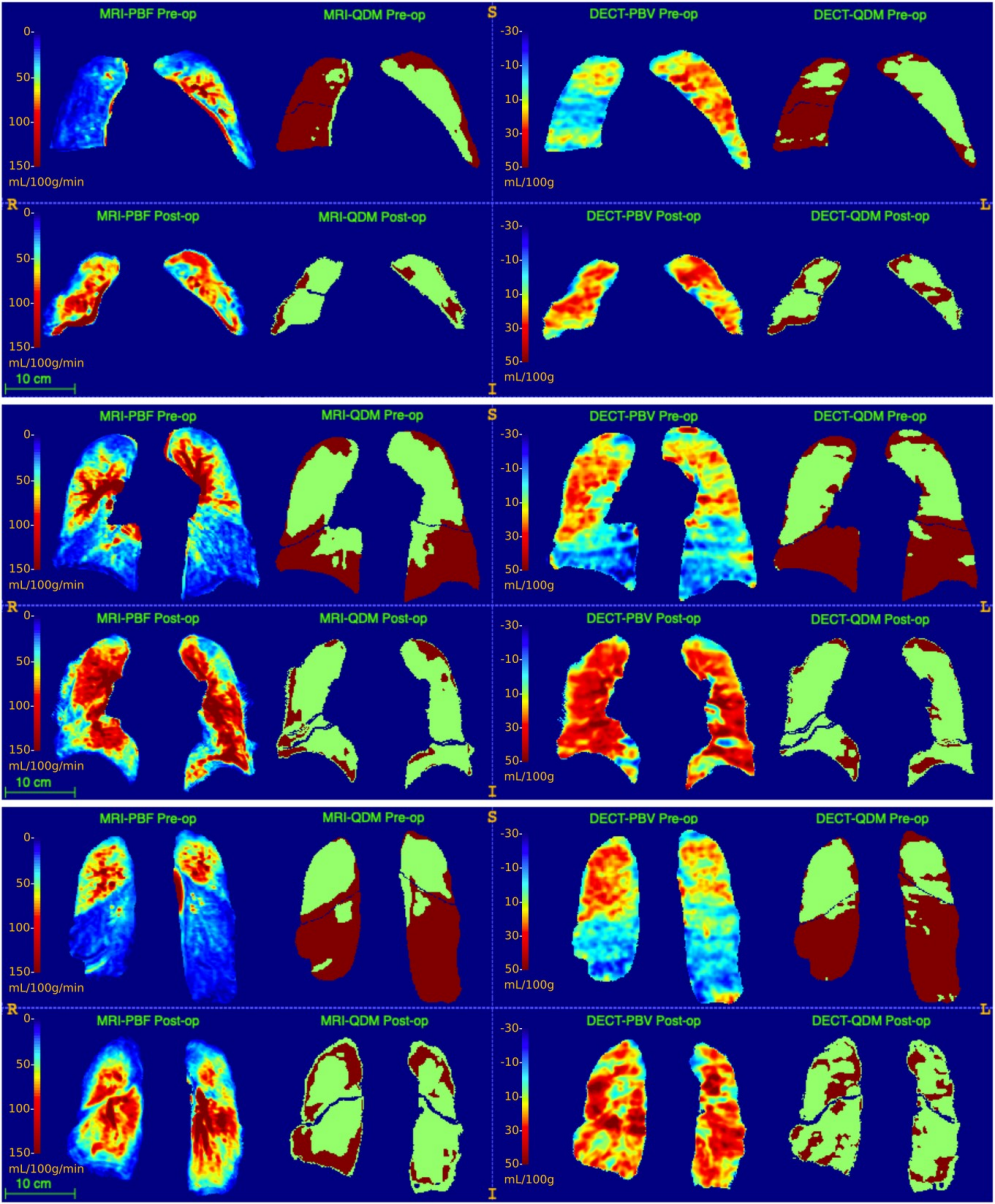
Sample coronal slices. These sample slices are taken from one study patient who completed all four scan sessions. The first and third columns from left show quantified maps of MRI-PBF and CT-PBV, respectively. On the right of each PBF or PBV map is its corresponding binary perfusion defect map. Odd rows are pre-op data sets while even rows are post-op. An obvious decrease in perfusion defects after PEA is seen primarily in lower lobes and in the middle lobe. A slightly smaller size of the lung is seen post-op compared to pre-op, probably due to post-op hypoventilation. In post-op MRI slices, marginal perfusion defects were detected and might be attributed to partial volume artifacts because of worse lung expansion compared to pre-op status.

In accordance with the improvement of mPAP and RVEF, a statistically significant increase in mean MRI-PBF and CT-PBV as well as a significant decrease in their corresponding QDP were measured (Tables 3 and 4, Fig 3). MRI-PBV showed no significant change post-op (S2 Table). Seven patients completed all DECT and MRI sessions and showed a mean decrease in CT-QDP of 9% (p = 0.097) and a mean decrease in MRI_(PBF)_-QDP of 7% (p = 0.015) (S3 Table). The mean difference between CT-QDP and MRI_(PBF)_-QDP in whole lung was 1.7% (SD 13.1%). After including additional 5 patients who completed pre- and post-op DECT scans (altogether 12 patients), a significant decrease in whole lung QDP of 8% was calculated (p = 0.007; Table 3). In overall 11 patients with completed pre- and post-op MRI scans, a significant decrease in whole lung MRI_(PBF)_-QDP of 7% was calculated (p = 0.004; Table 4).

**Table 3:** CT-PBV and CT-QDP changes in 12 patients after PEA.

ROI	CT-PBV) mL/100g(			Paired t-test (p-value)	CT-QDP (%)			Paired t-test (p-value)
	Pre-op	Post-op	Δ		Pre-op	Post-op	Δ	
**Whole lung**	14 (10)	23 (8)	+10 (7)	**0.001**	53 (8)	45 (6)	-8 (9)	**0.009**
**Right upper lobe**	15 (12)	22 (11)	+7 (7)	**0.003**	52 (20)	49 (18)	-3 (11)	0.391
**Right middle lobe**	8 (8)	17 (9)	+10 (10)	**0.006**	71 (15)	59 (17)	-12 (21)	0.079
**Right lower lobe**	11 (11)	27 (9)	+17 (11)	**<0.001**	64 (18)	36 (12)	-29 (23)	**0.001**
**Left upper lobe**	17 (11)	22 (8)	+4 (10)	0.163	42 (15)	49 (11)	+7 (15)	0.127
**Left lower lobe**	13 (10)	26 (11)	+13 (10)	**0.001**	56 (17)	40 (12)	-16 (20)	**0.020**

Listed values (besides p-values) are group means with the standard deviation in brackets. Bold p-values denote statistical significance (α = 0.05). CT-PBV: pulmonary blood volume estimated using dual-energy CT. CT-QDP: perfusion defect percentage calculated from CT-PBV.

**Table 4:** MRI-PBF and MRI-QDP improvement in 11 patients after PEA.

ROI	MRI-PBF (mL/100g/min)			Paired t-test (p-value)	MRI_(PBF)_-QDP (%)			Paired t-test (p-value)
	Pre-op	Post-op	Δ		Pre-op	Post-op	Δ	
**Whole lung**	46 (21)	64 (30)	+19 (20)	**0.013**	52 (4)	45 (7)	-7 (6)	**0.004**
**Right upper lobe**	49 (29)	64 (36)	+15 (21)	**0.038**	48 (17)	44 (15)	-4 (13)	0.398
**Right middle lobe**	44 (26)	60 (34)	+15 (27)	0.090	57 (13)	53 (12)	-5 (18)	0.396
**Right lower lobe**	41 (27)	70 (33)	+28 (29)	**0.009**	59 (19)	37 (11)	-22 (20)	**0.004**
**Left upper lobe**	49 (18)	59 (28)	+9 (21)	0.172	44 (13)	51 (10)	+7 (13)	0.108
**Left lower lobe**	39 (18)	69 (28)	+30 (23)	**0.002**	63 (15)	40 (15)	-23 (18)	**0.001**

7 mutual patients with the analysis represented in Table 3. Bold p-values denote statistical significance (α = 0.05). Values are the statistical means with the standard deviation in brackets. MRI-PBF: pulmonary blood flow estimated from dynamic contrast-enhanced MRI. MRI_(PBF)_-QDP: Perfusion defect percentage calculated from MRI-PBF.

On lobar level and as estimated from both modalities, lung perfusion was mostly improved in right and left lower lobes with QDP decrease of 22–29% (right lower) and 16–23% (left lower). Using CT-PBV and MRI-PBF, no change in the estimated QDP was detected in the left upper lobe (7%; p > 0.108), however, MRI_(PBV)_-QDP showed an increase of 9% (p = 0.043).

## Discussion/Conclusions

In this work, we compared dual-energy CT to dynamic contrast-enhanced MRI in evaluating lung perfusion defects in chronic thromboembolic pulmonary hypertension. Both imaging modalities detected significantly correlating perfusion defect percentages with a good spatial agreement between defect maps. CT and MRI detected similar and statistically significant decrease in perfusion defects after pulmonary endarterectomy.

Based on our results, DECT may be regarded as a non-inferior alternative to MRI in assessing the severity of CTEPH and in monitoring patients after surgical and interventional therapies. Despite radiation exposure, CT could be more practical especially in post-operative settings, besides offering the possibility to scan patients with contraindications to MRI. In addition, CT has the advantage of high spatial resolution allowing it to detect other comorbidities such as chronic obstructive pulmonary disease and small lung nodules. MRI, on the other hand, allows also the assessment of perfusion dynamics and cardiac function in a single “one stop shop” examination.

Previous studies have already shown that a significant reduction in lung perfusion defects is expected after pulmonary endarterectomy with concomitant reduction in mPAP and improvement in right heart function. Our lobe-based results show that the postoperative improvement in lung perfusion is primarily noticeable in lower lung lobes. Arteries in the lower lobes are thought to be more affected than those in the upper lobes are [15, 25], hence profit more from therapy. MRI and CT, however, showed a similar contribution of the lower and upper lobes to pre-op whole lung defect percentage (S4 Table). The reason might be an underestimation of the defect percentage in lower lobes because of excluded basal areas (remarkable motion artefacts from diaphragm in MRI) and pericardial areas (motion and partial volume artefacts in MRI as well as CT beam hardening artefacts from the presence of high-density iodine contrast agent in the heart champers). The left upper lobe showed in both imaging modalities a mild increase in the defect percentage post-op. However, this difference was only significant in the smaller patient collective (7 patients) or when calculated from MRI-PBV. The reason for this might be a blood flow normalization post-op due to a redistribution of pulmonary flow with higher flow in re-perfused lower lobes capillaries.

Although no significant correlation was found between the absolute values of CT-PBV (derived from DECT iodine maps) and MRI-PBF and MRI-PBV (calculated from DCE-MRI), DECT provided similar QDP estimates in CTEPH patients compared to DCE-MRI (in Bland-Altman plots in Figs 1 and 2 the differences in QDP for each subject generally fell within the 95% limits of agreement). This could be explained by the dependency of the absolute pulmonary iodine concentration on cardiac function while on the other hand, DCE-MRI provides a direct, fully quantifiable measure of the blood flow.

The main limitation of this study was the low number of patients who completed all imaging sessions. However, a relatively good number of CT-MRI pairs was acquired which allowed to compare both modalities in terms of estimating QDP. Calculated parameters were compared to each other and not to a gold standard. Reference imaging procedures such as lung scintigraphy or pulmonary angiography were not indicated in the study cohort because the patients were already diagnosed with CTEPH. Another limitation was that no motion correction of the DCE-MRI data sets was performed as the images were acquired in breath hold and hence expected to have no or minimal motion artifacts. In some PBF maps, distinct ribbon-like artifacts from diaphragmatic motion could be identified in basal lung regions. These areas were then excluded from the lung segmentation with the probable consequence of a non-accurate defect quantification in the lower lobes.

To conclude, DECT and DCE-MRI provided a comparable quantification of lung perfusion defects in CTEPH patients with a good spatial agreement in defect maps. It was also shown that both imaging modalities could be used to assess therapeutic success.

## Supporting information

S1 Table Correlation analysis of the absolute values of CT-PBV with MRI-PBF and with MRI-PBV. Listed values are means from 19 patients (standard deviation in brackets).
(DOCX)

S2 Table Mean MRI-PBV and QDP improvement in 11 patients after PEA (7 common patients with the analysis represented in Table 2). Bold p-values denote statistical significance (α = 0.05). Standard deviation in brackets.
(DOCX)

S3 Table Whole lung and lobe based QDP changes in 7 patients after PEA as estimated from DECT (left columns) and DCE-MRI (right columns). Bold p-values denote statistical significance (α = 0.05). Listed values are means with corresponding standard deviations in brackets.
(DOCX)

S4 Table Lobe based CT- and MRI_(PBF)_-QDP calculated in reference to whole lung volume. The two right columns show Pearson correlation coefficients and corresponding p-values (bold p-values denote statistical significance α = 0.05).
(DOCX)

S1 Dataset Pulmonary blood flow und volume parameters in whole lung and in each lobe as estimated from both imaging modalities. (XLSX)
