# Supplementary material for: Comparison of dual-energy computer tomography and dynamic contrast-enhanced MRI for evaluating lung perfusion defects in chronic thromboembolic pulmonary hypertension
Source: PLoS One. 2021 Jun 17;16(6):e0251740. doi: 10.1371/journal.pone.0251740 (PMC8211171; doi:10.1371/journal.pone.0251740)
Supplement: S1 Table — Listed values are means from 19 patients (standard deviation in brackets). (DOCX) [file pone.0251740.s001.docx]

S1 Table: Correlation analysis of the absolute values of CT-PBV with MRI-PBF and with MRI-PBV. Listed values are means from **19 patients** (standard deviation in brackets).

| **ROI** | **CT-PBV (mL/100g(** | **MRI-PBF (mL/100g/min)** | **Pearson correlation**  **CT-PBV / MRI-PBF** | | **MRI-PBV (mL/100g(** | **Pearson correlation**  **CT-PBV / MRI-PBV** | |
| --- | --- | --- | --- | --- | --- | --- | --- |
|  |  |  | **r** | **p-value** |  | **r** | **p-value** |
| **Whole lung** | 19 (8) | 53 (23) | 0.13 | 0.602 | 7 (3) | 0.08 | 0.750 |
| **Right upper lobe** | 22 (11) | 57 (30) | 0.43 | 0.063 | 7 (3) | 0.28 | 0.245 |
| **Right middle lobe** | 14 (10) | 48 (26) | 0.39 | 0.096 | 7 (4) | 0.25 | 0.306 |
| **Right lower lobe** | 18 (12) | 52 (29) | 0.44 | 0.057 | 8 (4) | 0.40 | 0.089 |
| **Left upper lobe** | 19 (8) | 54 (23) | 0.13 | 0.607 | 7 (3) | 0.22 | 0.372 |
| **Left lower lobe** | 15 (10) | 49 (19) | 0.02 | 0.930 | 7 (4) | -0.10 | 0.679 |

CT-PBV: pulmonary blood volume estimated using dual-energy CT. MRI-PBF: pulmonary blood flow estimated from dynamic contrast-enhanced MRI.
