# Supplementary material for: Comparison of dual-energy computer tomography and dynamic contrast-enhanced MRI for evaluating lung perfusion defects in chronic thromboembolic pulmonary hypertension
Source: PLoS One. 2021 Jun 17;16(6):e0251740. doi: 10.1371/journal.pone.0251740 (PMC8211171; doi:10.1371/journal.pone.0251740)
Supplement: S2 Table — Bold p-values denote statistical significance (α = 0.05). Standard deviation in brackets. (DOCX) [file pone.0251740.s002.docx]

S2 Table: Mean MRI-PBV and QDP improvement in **11 patients** after PEA (7 common patients with the analysis represented in Table 2). Bold p-values denote statistical significance (α = 0.05). Standard deviation in brackets.

| **ROI** | **MRI-PBV (mL/100g(** | | | **Paired  t-test (p-value)** | **MRI_(PBV)_-QDP (%)** | | | **Paired  t-test (p-value)** |
| --- | --- | --- | --- | --- | --- | --- | --- | --- |
|  | **Pre-op** | **Post-op** | **Δ** |  | **Pre-op** | **Post-op** | **Δ** |  |
| **Whole lung** | 10 (6) | 7 (4) | -3 (6) | 0.157 | 47 (9) | 47 (8) | 0 (6) | 0.918 |
| **Right upper lobe** | 9 (4) | 7 (3) | -2 (4) | 0.117 | 53 (14) | 48 (16) | -5 (13) | 0.226 |
| **Right middle lobe** | 8 (4) | 7 (3) | -1 (4) | 0.317 | 59 (14) | 53 (13) | -6 (15) | 0.195 |
| **Right lower lobe** | 11 (7) | 7 (5) | -5 (9) | 0.121 | 36 (12) | 49 (20) | +13 (19) | **0.046** |
| **Left upper lobe** | 9 (6) | 7 (3) | -2 (6) | 0.369 | 53 (11) | 44 (12) | -9 (12) | **0.043** |
| **Left lower lobe** | 12 (8) | 6 (5) | -6 (11) | 0.112 | 35 (15) | 49 (16) | +14 (17) | **0.019** |

MRI-PBV: pulmonary blood volume estimated from dynamic contrast-enhanced MRI. MRI**_(PBV)_**-QDP: perfusion defect percentage calculated from MRI-PBV maps.
