# Supplementary material for: Comparison of dual-energy computer tomography and dynamic contrast-enhanced MRI for evaluating lung perfusion defects in chronic thromboembolic pulmonary hypertension
Source: PLoS One. 2021 Jun 17;16(6):e0251740. doi: 10.1371/journal.pone.0251740 (PMC8211171; doi:10.1371/journal.pone.0251740)
Supplement: S3 Table — Bold p-values denote statistical significance (α = 0.05). Listed values are means with corresponding standard deviations in brackets. (DOCX) [file pone.0251740.s003.docx]

S3 Table: Whole lung and lobe based QDP changes in **7 patients** after PEA as estimated from DECT (left columns) and DCE-MRI (right columns). Bold p-values denote statistical significance (α = 0.05). Listed values are means with corresponding standard deviations in brackets.

| **ROI** | **CT-QDP**  **(%)** | | | **Paired  t-test (p-value)** | **MRI_(PBF)_-QDP**  **(%)** | | | **Paired  t-test (p-value)** |
| --- | --- | --- | --- | --- | --- | --- | --- | --- |
|  | **Pre-op** | **Post-op** | **Δ** |  | **Pre-op** | **Post-op** | **Δ** |  |
| **Whole lung** | 53 (9) | 46 (8) | -7 (10) | 0.097 | 53 (3) | 44 (8) | -9 (7) | **0.015** |
| **Right upper lobe** | 54 (17) | 46 (12) | -8 (11) | 0.101 | 55 (15) | 45 (16) | -10 (13) | 0.084 |
| **Right middle lobe** | 65 (14) | 57 (19) | -8 (24) | 0.414 | 61 (13) | 52 (11) | -10 (20) | 0.258 |
| **Right lower lobe** | 65 (17) | 37 (14) | -28 (25) | **0.025** | 63 (16) | 37 (13) | -26 (17) | **0.007** |
| **Left upper lobe** | 40 (16) | 53 (10) | +13 (12) | **0.029** | 41 (13) | 51 (13) | +11 (12) | 0.062 |
| **Left lower lobe** | 57 (18) | 39 (10) | -17 (23) | 0.087 | 58 (14) | 36 (13) | -22 (21) | **0.033** |

CT-QDP and MRI**_(PBF)_**-QDP: perfusion defect percentage calculated from dual-energy CT based and MRI based PBF maps, respectively.
