# Supplementary material for: Comparison of dual-energy computer tomography and dynamic contrast-enhanced MRI for evaluating lung perfusion defects in chronic thromboembolic pulmonary hypertension
Source: PLoS One. 2021 Jun 17;16(6):e0251740. doi: 10.1371/journal.pone.0251740 (PMC8211171; doi:10.1371/journal.pone.0251740)
Supplement: S4 Table — The two right columns show Pearson correlation coefficients and corresponding p-values (bold p-values denote statistical significance α = 0.05). (DOCX) [file pone.0251740.s004.docx]

S4 Table: Lobe based CT- and MRI**_(PBF)_**-QDP calculated in reference to whole lung volume. The two right columns show Pearson correlation coefficients and corresponding p-values (bold p-values denote statistical significance α = 0.05).

| **ROI** | **CT-QDP**  **(%)** | **MRI_(PBF)_-QDP**  **(%)** | **Pearson correlation coefficient** | |
| --- | --- | --- | --- | --- |
|  |  |  | **r** | **p-value** |
| **Whole lung** | 47 (7) | 50 (5) | 0.51 | **0.026** |
| **Right upper lobe** | 9 (4) | 11 (3) | 0.64 | **0.003** |
| **Right middle lobe** | 7 (2) | 7 (2) | 0.71 | **0.001** |
| **Right lower lobe** | 9 (4) | 10 (4) | 0.73 | **<0.001** |
| **Left upper lobe** | 11 (4) | 13 (4) | 0.85 | **<0.001** |
| **Left lower lobe** | 10 (4) | 10 (4) | 0.81 | **<0.001** |

CT-QDP and MRI_(PBF)_-QDP: perfusion defect percentage calculated from dual-energy CT based and MRI based PBF maps, respectively.
